# Pre-hospital admission of heparin in patients with suspected non-ST segment elevation acute coronary syndrome

**DOI:** 10.1007/s00392-024-02507-1

**Published:** 2024-08-05

**Authors:** Jonas Sundermeyer, Alina Schock, Caroline Kellner, Paul M. Haller, Jonas Lehmacher, Niklas Thießen, Betül Toprak, Lea Scharlemann, Raphael Twerenbold, Nils Arne Sörensen, Peter Clemmensen, Johannes T. Neumann

**Affiliations:** 1 Department of Cardiology, University Heart and Vascular Center Hamburg, Martinistr. 52, 20251 Hamburg, Germany; 2 German Center for Cardiovascular Research (DZHK), Partner Site Hamburg/Kiel/Lübeck, Hamburg, Germany; 3 Center for Population Health Innovation (POINT), Hamburg, Germany

**Keywords:** Acute coronary syndrome, NSTE-ACS, Heparin, Pre-hospital

## Abstract

**Background:**

Evidence supporting pre-hospital heparin administration in patients with suspected non-ST segment elevation acute coronary syndrome (NSTE-ACS) is lacking. We aim to evaluate if pre-hospital heparin administration by emergency medical service improves clinical outcome in patients with suspected NSTE-ACS.

**Methods:**

Patients with suspected myocardial infarction (MI) presenting to the emergency department were prospectively enrolled from 2013 to 2021, excluding those with ST segment elevation MI. Patients with and without prehospital heparin administration were compared using propensity score matching. To assess the association between pre-hospital heparin loading, 30-day and 1-year mortality, Kaplan–Meier estimations and Cox regression models were used.

**Results:**

Among 1,234 patients, median age was 69 years, 755 (61.2%) were male, 867 (70.5%) with known hypertension, 177 (14.4%) had diabetes, 280 (23.1%) were current smokers, and 444 (36.0%) had a history of CAD. Compared to patients without pre-hospital heparin administration, heparin pre-treated patients were more often active smokers (26.5% vs. 20.8%). After propensity matching, 475 patients with vs. without pre-hospital heparin administration were compared, with no significant difference in 30-day mortality (no-heparin 1.3% vs. heparin 0.4%) and 1-year mortality (no-heparin 7.2% vs. heparin 5.5%, adjusted HR 0.98, CI 0.95–1.01, *p* = 0.22). Bleeding events occurred at a low frequency (< 2%) and did not differ between groups.

**Conclusions:**

In this study, pre-hospital heparin administration was not associated with improved clinical outcome in patients with suspected NSTE-ACS. These findings question pre-hospital heparin therapy in this patient population and might potentially warrant a more restricted utilization pending in-hospital risk assessment.

**Graphical abstract:**

Pre-hospital admission of heparin in patients with suspected non-ST segment elevation acute coronary syndrome. *ACS* acute coronary syndrome, *CI* confidence interval, *HR* hazard ratio, *NSTE* non-ST segment elevation, *STEMI* ST-elevation myocardial infarction, *UFH* unfractionated heparin.
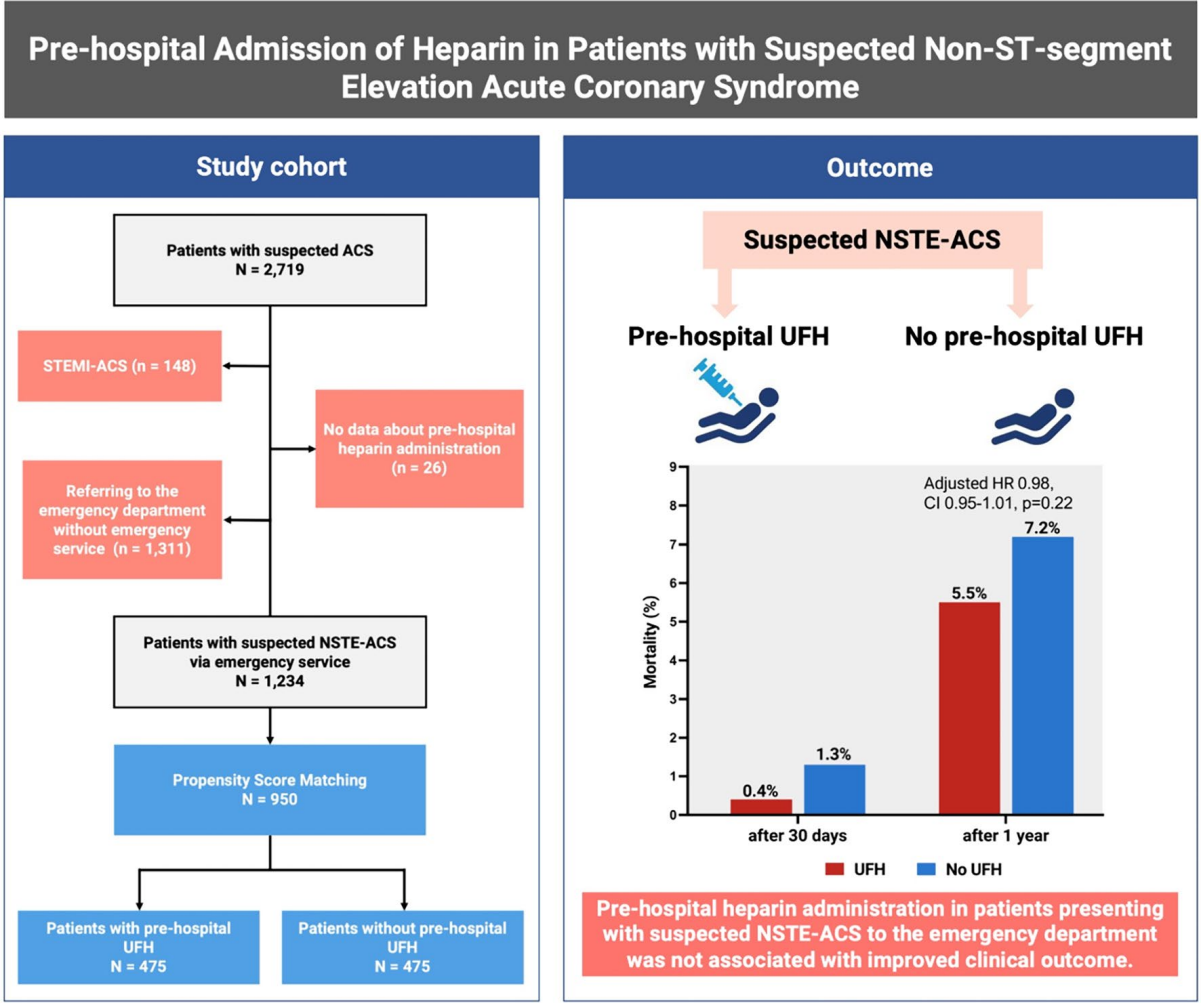

**Supplementary Information:**

The online version contains supplementary material available at 10.1007/s00392-024-02507-1.

## Introduction

Non-ST segment elevation acute coronary syndromes (NSTE-ACS), encompassing non-ST-elevation myocardial infarction (NSTEMI) and unstable angina, represent one of the primary reasons for consultations in emergency departments, with an increasing frequency [1–4].

The preclinical basic care for myocardial infarction (MI) is well-established in emergency medical services and patients suspected of having NSTE-ACS are regularly transported to the emergency department [1, 5, 6]. However, there is still a notable lack of evidence supporting the use of medication before hospital admission, particularly regarding parenteral anticoagulation in these MI patients [1]. Previous studies have suggested that the administration of parenteral anticoagulation is linked to a reduced risk of adverse cardiovascular events in individuals diagnosed with NSTE-ACS [7, 8]. Heparin is commonly administered during percutaneous coronary intervention (PCI) [1, 5, 6]. However, analyses regarding the benefits of heparin were primarily conducted before the widespread adoption of dual antiplatelet therapy and PCI as the state-of-the-art management in acute MI. Specifically, the superiority of preclinical administration of anticoagulants over their administration following in-hospital confirmation of NSTEMI diagnosis remains uncertain [9]. Consequently, it varies in pre-hospital settings.

Therefore, there is an ongoing uncertainty regarding the efficacy and safety of preclinical parenteral anticoagulation in patients with suspected NSTE-ACS. Our study hypothesis aims to evaluate whether restrictive pre-hospital heparin administration before in-hospital risk assessment is non-inferior to pre-hospital heparin loading by emergency medical services in patients with suspected NSTE-ACS. Clinical outcomes and safety were assessed in a large prospective cohort of patients with symptoms suggestive of acute MI.

## Methods

### Data source and setting

This analysis was conducted in the Biomarkers in Acute Cardiac Care (BACC) study, an ongoing, single-center observational cohort study (NCT02355457). Detailed information about data entry, index event definition and inclusion/exclusion criterions has been previously reported [3, 10–12]. In summary, this study prospectively enrolls patients with symptoms suggestive of acute MI and presenting to the emergency department of the University Medical Center Hamburg-Eppendorf were prospectively enrolled. These patients received treatment in accordance with the standards of the University Medical Center Hamburg-Eppendorf and respective guidelines [1, 13]. As part of the standard emergency care to guide subsequent diagnostic and therapeutic approaches, patients underwent an electrocardiogram, routine laboratory assessments, including high-sensitivity cardiac troponin T (hs-cTnT) measurements at presentation (0 h), 1 and 3 h, as well as additional imaging as appropriate. All decisions were at the discretion of the treating physician and with the exception of sample collection for hs-cTn determination were not mandated by the study.

For the present project, we restricted our analyses to patients who have been enrolled between 2013 and 2019. The study adheres to the principles of the Declaration of Helsinki and received approval from the local ethics committees. Written informed consent was obtained from all patients. The primary data supporting the study findings can be obtained from the corresponding author upon a reasonable request.

### Follow-up

Patients were followed for up to 8 years using a census-based system. Study personnel, trained for this purpose, conducted structured interviews via telephone to collect information on various events of interest, such as all-cause mortality, cardiovascular death, acute MI, unplanned revascularization, cardiac re-hospitalization, and cardiac symptoms. In cases where direct contact was challenging (mail, E-mail, communication via family physicians), and if information remained elusive, the local death registry was consulted to retrieve details on potential deaths.

### Definition of study groups

Patients with ST-elevation myocardial infarction (STEMI) were excluded from this study, as illustrated in the *Graphical Abstract*. Only patients with suspected NSTE-ACS referring to the emergency department via emergency service were further categorized based on pre-hospital unfractionated heparin (UFH) administration vs. no pre-hospital UFH administration. UFH (5.000 I.E.) was the sole parenteral anticoagulant administered in the preclinical setting in this cohort. Acetylsalicylic acid at a dosage of 250 mg was routinely part of the pre-hospital protocol. There was no pre-hospital administration of P2Y12 inhibitors during the study period. Documentation is standardized in the work flow for the handover of patients from physicians of the emergency medical services to those in the emergency department. For patients with an urgent indication for angiography, determined by treating physicians during the initial in-hospital assessment, heparin was administered promptly within the first 10 min after reaching the hospital.

### Outcome

The primary outcome of this study was the cumulative all-cause mortality at 30 days and 1 year. Bleeding events were assessed as secondary outcomes, with ICD-10 code query for the following codes: I60, I61, I62, S06.3, S06.4, S06.5, S06.6, I85.0, K25.0, K25.2, K25.4, K25.6, K26.0, K26.2, K26.4, K26.6, K27.0, K272, K27.4, K27.6, K28.0, K282, K28.4, K28.6, K29.0, K62.5, K92.0, K92.1, K92.2, D62, N02, R31, R58, H11.3, H35.6, H43.1, H45.0, H92.2, J94.2, K66.1, M25.0, N92.0, N92.1, N92.4, N93.8, N93.9, N95.0, R04.0, R04.1, R04.2, R04.8, R04.9. All secondary endpoints were in-hospital events, and data regarding bleeding events beyond discharge were not collected during follow-up.

### Statistical analyses

Continuous variables were described using the median (25th percentile, 75th percentile) and analyzed using the Kruskal–Wallis test. Binary variables were presented in terms of absolute and relative frequencies, and comparisons were conducted using chi-square test.

To investigate differences in clinical characteristics and comorbidities during the index event between patients with pre-hospital UFH administration vs. no pre-hospital UFH administration, multivariable mixed effects logistic regression models with center as a random intercept were fitted, adjusted for sex and MI diagnoses.

A propensity score matched analysis was performed to compare the outcome of patients with suspected NSTE-ACS with pre-hospital UFH administration vs. no pre-hospital UFH administration. The identified matching variables encompassed sex, age, hypertension, current smoking, history of coronary artery disease, bypass or percutaneous coronary intervention, ischemic ECG, pre-existing anticoagulation, and pre-existing medication with acetylsalicylic acid. Using these covariates, propensity scores were calculated for each patient. Based on these propensity scores, patients pre-treated preclinically with UFH vs. no UFH were matched 1:1 using the optimal pair matching method with Mahalanobis distance measure and no replacement. The balance in potential confounders between the study groups was evaluated based on the average absolute standardized difference and amounts 0.121 before and 0.020 after matching. A threshold of 0.10 was set, deeming values below this threshold as not statistically significant. This finally resulted in 475 propensity matched pairs.

The duration from the baseline to death was used to calculate the 30-day and 1-year mortality. Crude mortality rates were estimated using the Kaplan–Meier method, with the number of individuals at risk reported. Group comparisons were performed using the log-rank test. To assess the association between pre-hospital UFH administration vs. no pre-hospital UFH administration, cohort stratified Cox proportional hazard regression models were fitted, adjusted for age, sex, and NSTEMI diagnoses during hospital course.

To assess the impact of pre-hospital UFH administration on mortality within pre-specified subgroups (e.g., confirmed NSTE-ACS vs. unconfirmed NSTE-ACS), Kaplan–Meier analysis, propensity score matching, and cohort stratified Cox proportional hazard regression models were fitted and adjusted as previously described.

A p value below 0.05 was deemed statistically significant. All analyses were performed with R statistical software version 4.1.2.

## Results

### Unmatched study cohort

A total of 1234 patients with suspected NSTE-ACS were enrolled and eligible for this study (*Graphical Abstract)*, of whom 503 (40.8%) were treated with and 731 (59.2%) without prehospital UFH administration (Table 1*)*.

**Table 1:** Characteristics for the unmatched and matched study cohort

	All (*N* = 1234)	Missings (%)	Unmatched study cohort			Matched study cohort		
			No UFH (*N* = 731)	UFH (*N* = 503)	*p* value	No UFH (*N* = 475)	UFH (*N* = 475)	*p* value
*Baseline characteristics*								
Age (years, IQR)	69.0 (55.0, 77.8)	0	70.0 (55.0, 78.0)	69.0 (56.0, 77.0)	0.87	69.0 (55.0, 77.0)	69.0 (56.0, 77.0)	0.67
Male no. (%)	755 (61.2)	0	431(59.0)	324 (64.4)	0.061	302 (63.6)	306 (64.4)	0.84
Median body mass index (kg/m2, IQR)	26.4 (23.7, 30.1)	11.6	26.3 (23.6, 30.2)	26.6 (23.7, 29.8)	0.90	26.6 (23.9, 30.1)	26.6 (23.8, 29.8)	0.94
History of hypertension no. (%)	867 (70.5)	0.3	515 (70.7)	352 (70.1)	0.86	340 (71.6)	337 (70.9)	0.89
History of diabetes no. (%)	177 (14.4)	0.7	103 (14.2)	74 (14.8)	0.84	69 (14.5)	70 (14.7)	1.00
History of hyperlipoproteinemia no. (%)	427 (34.6)	0	241 (33.0)	186 (37.0)	0.16	170 (35.8)	175 (36.8)	0.79
Former smoker no. (%)	284 (23.5)	1.9	172 (24.0)	112 (22.7)	0.64	110 (23.2)	107 (22.5)	0.88
Current smoking no. (%)	280 (23.1)	1.9	149 (20.8)	131 (26.5)	0.024	121 (25.5)	124 (26.1)	0.88
History of CAD no. (%)	444 (36.0)	0	248 (33.9)	196 (39.0)	0.080	176 (37.1)	184 (38.7)	0.64
History of stroke no. (%)	98 (7.9)	0.1	56 (7.7)	42 (8.3)	0.74	33 (7.0)	39 (8.2)	0.55
History of peripheral artery disease no. (%)	77 (6.2)	0	46 (6.3)	31 (6.2)	1.00	30 (6.3)	28 (5.9)	0.89
CKD (eGFR < 60 ml/min) No. (%)	420 (34.3)	0.6	507 (25.1)	164 (32.7)	0.38	163 (34.5)	152 (32.1)	0.49
Prior oral anticoagulation	232 (19.0)	1.0	170 (23.5)	62 (12.4)	< 0.001	60 (12.6)	59 (12.4)	1.00
Prior acetylic salicylic acid	469 (38.3)	0.9	248 (34.3)	221 (44.3)	< 0.001	200 (42.1)	208 (43.8)	0.65
Ischemia signs on ECG no. (%)	286 (24.0)	3.3	164 (23.3)	122 (25.0)	0.53	122 (25.7)	121 (25.5)	1.00
Symptom onset < 3 h before presentation no. (%)	467 (40.1)	5.6	261 (38.1)	206 (42.9)	0.11	169 (37.9)	192 (42.2)	0.21
Symptom onset ≥ 3 h before presentation no. (%)	698 (59.9)	5.6	424 (61.9)	274 (57.1)	0.11	277 (62.1)	263 (57.8)	0.21
Laboratory measurements								
Median eGFR (mL/min for 1.73m^2^, IQR)	72.8 (53.2, 88.9)	0.6	73.1 (52.0, 89.3)	72.3 (54.9, 88.1)	0.79	73.5 (53.3, 89.8)	72.8 (55.2, 88.3)	0.91
Median high-sensitivity troponin I (ng/l IQR)								
At emergency department presentation	6.9 (3.0, 23.8)	4.2	6.8 (2.9, 22.3)	7.4 (3.2, 25.5)	0.20	6.9 (3.1, 24.8)	7.2 (3.3, 25.4)	0.60
At 60 min	7.6 (3.0, 30.2)	4.4	7.0 (2.9, 25.3)	8.8 (3.2, 46.4)	0.044	7.4 (3.1, 27.0)	8.8 (3.2, 45.1)	0.25
At 180 min	8.7 (3.6, 38.7)	3.7	8.1 (3.5, 30.4)	9.9 (3.8, 61.4)	0.023	8.7 (3.6, 32.0)	9.8 (3.8, 61.3)	0.18
*Cardiac examinations during hospital course*								
Angiography no. (%)	350 (28.4)	0	157 (21.5)	193 (38.4)	< 0.001	107 (22.5)	180 (37.9)	< 0.001
Revascularization no. (%)	181 (14.7)	0	81 (11.1)	100 (19.9)	< 0.001	61 (12.8)	96 (20.2)	0.003
Final diagnosis of NSTEMI no. (%)	238 (19.3)	0	113 (15.5)	125 (24.9)	< 0.001	77 (16.2)	120 (25.3)	< 0.001
TTE LV ejection fraction								
Normal no. (%)	773 (76.8)	18.4	416 (76.5)	357 (77.1)	0.87	279 (76.2)	338 (77.0)	0.86
Mildly reduced no. (%)	100 (9.9)	18.4	53 (9.7)	47 (10.2)	0.91	39 (10.7)	46 (10.5)	1.00
Severely reduced no. (%)	69 (6.9)	18.4	31 (5.7)	38 (8.2)	0.15	28 (7.7)	20 (4.6)	0.090

Continuous variables are shown as a median (25th, 75th percentile), the *p* value given is calculated using the Kruskal–Wallis test. Binary variables are shown as absolute and relative frequencies, the *p* value given is calculated by chi-square test

*CAD* coronary artery disease, *CKD* chronic kidney disease, *ECG* electrocardiogram, *eGFR* estimated glomerular filtration rate, *NSTEMI* non-ST segment elevation myocardial infarction, *OAC* oral anticoagulation, *PCI* percutaneous coronary intervention, *TTE* transthoracic echocardiogram, *LV* left ventricular, *UFH* unfractionated heparin

In the unmatched study cohort, median age was 69 [interquartile range (IQR) 55–78] years and 755 (61.2%) of the patients were male. A total of 867 (70.5%) patients presented with known hypertension, 177 (14.4%) had diabetes, and 280 (23.1%) were current smokers. Overall, 444 (36.0%) patients had a known history of CAD (Table 1).

**Fig. 1:**
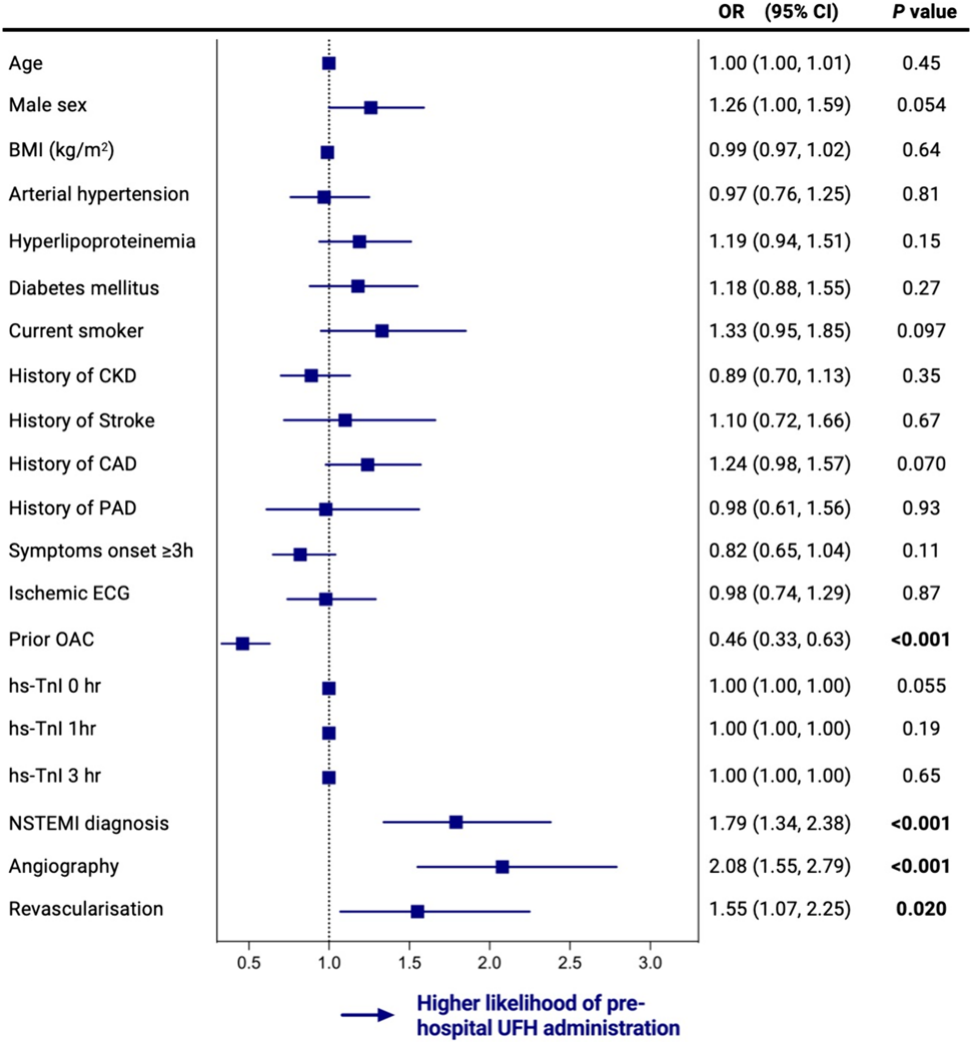
Association between characteristics of patients with NSTE-ACS and prehospital heparin administration vs. no prehospital heparin administration. Odds ratio calculated by multivariable mixed effects logistic regression for no pre-hospital UFH administration vs. pre-hospital UFH administration in patients with NSTE-ACS. *BMI* body mass index, *CAD* coronary artery disease, *CI* confidence interval, *CKD* chronic kidney disease, *ECG* electrocardiogram, *NSTE-ACS* non-ST segment elevation acute coronary syndromes, *NSTEMI* non-ST segment elevation myocardial infarction, *OR* odds ratio, *OAC* oral anticoagulation, *PAD* peripheral artery disease, *UFH* unfractionated heparin

Compared to patients without pre-hospital UFH administration, those pre-treated with UFH in the unmatched cohort were more often active smokers (26.5% vs. 20.8%), as illustrated in Table 1*.* After adjustment for relevant confounders, cardiovascular risk factors, such as age, sex, hypertension, diabetes, hyperlipoproteinemia, current smoking status, history of CAD, chronic kidney disease (CKD) or stroke, were not independently associated with pre-hospital UFH administration (Fig. 1). Patients without prehospital UFH administration were more likely to have had oral anticoagulation in their premedication (OR 0.46, 95% CI 0.33–0.63, *p* < 0.001, Fig. 1).

During the index hospital course, patients with pre-hospital UFH administration more likely received coronary angiography (OR 2.08, 95% CI 1.55–2.79, *p* < 0.001) and percutaneous coronary intervention (OR 1.55, 95% CI 1.07–2.25, *p* = 0.020) and were more frequently diagnosed with intrahospital confirmed NSTEMI (OR 1.79, 95% CI 1.34–2.38, *p* < 0.001, Fig. 1).

### Matched study cohort

After propensity matching, 475 patients with pre-hospital UFH administration were compared to 475 patients without pre-hospital UFH administration. Baseline characteristics of the matched study cohort are illustrated in Table 1. The distribution of matching characteristics between both groups was effectively balanced, including age (no pre-hospital UFH 69 years vs. pre-hospital UFH 69 years), male sex (no pre-hospital UFH 63.6% vs. pre-hospital UFH 64.4%), hypertension (no pre-hospital UFH 71.6% vs. pre-hospital UFH 70.9%), current smoking (no pre-hospital UFH 25.5% vs. pre-hospital UFH 26.1%), history of CAD (no pre-hospital UFH 37.1% vs. pre-hospital UFH 38.7%), ischemic ECG (no pre-hospital UFH 25.7% vs. pre-hospital UFH 25.5%), prior intake of anticoagulant (no pre-hospital UFH 12.6% vs. pre-hospital UFH 12.4%), and prior acetylsalicylic acid medication (no pre-hospital UFH 42.1% vs. pre-hospital UFH 43.8%, Table 1*)*. Furthermore, no significant differences were observed in terms of body mass index, hyperlipoproteinemia, diabetes, prior history of stroke, chronic kidney disease, or peripheral artery disease (Table 1).

### Association between pre-hospital heparin administration, mortality, and safety endpoints

**Fig. 2:**
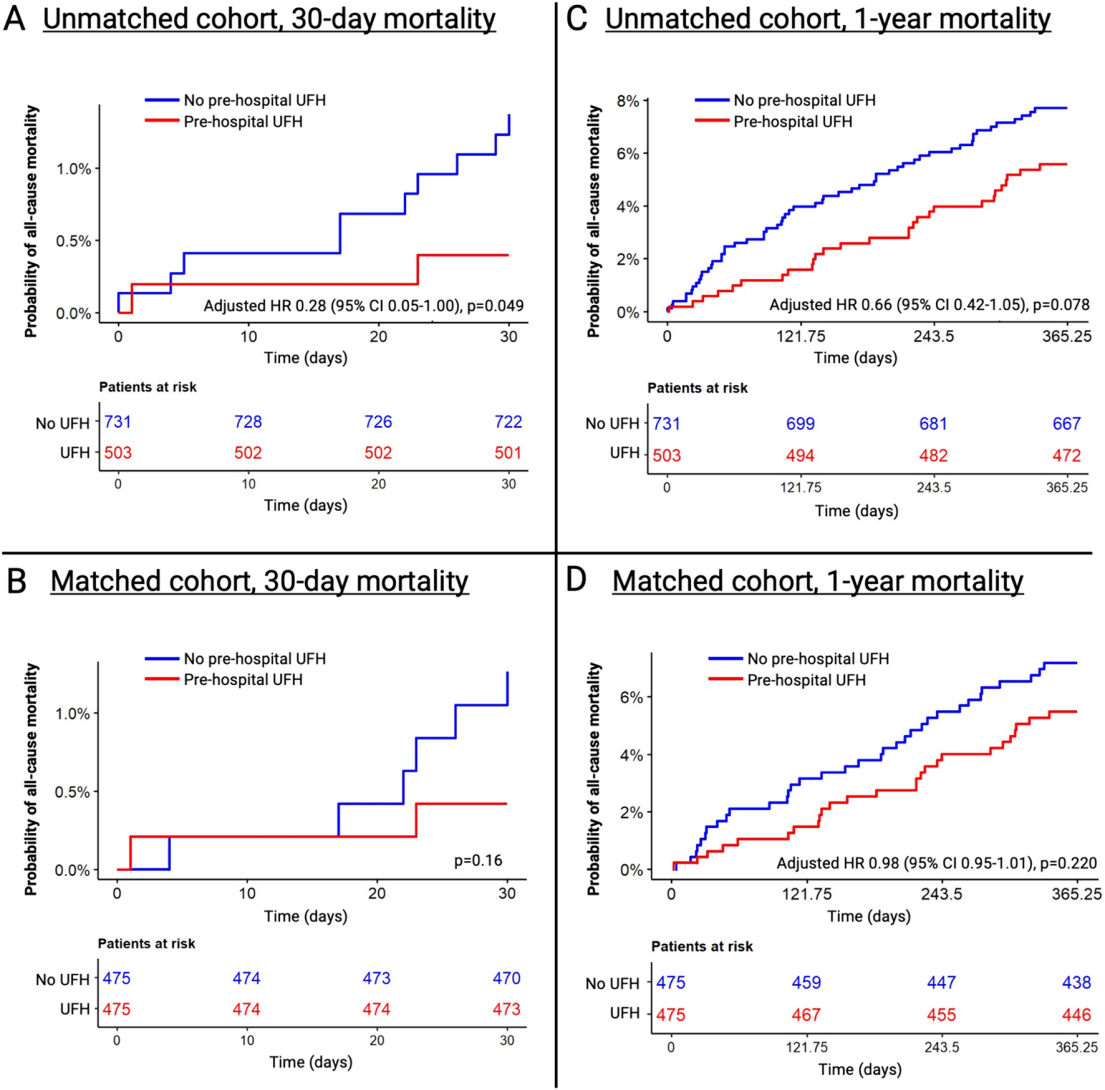
Kaplan–Meier curves of the unmatched and matched study cohort comparing patients with NSTE-ACS and pre-hospital UFH administration vs. without pre-hospital UFH administration. Kaplan–Meier curves of the unmatched and matched study cohort comparing patients with suspected NSTE-ACS treated with versus without pre-hospital UFH. **A** 30-day mortality of the unmatched cohort. **B** 30-day mortality of the matched cohort. **C** 1-year mortality of the unmatched cohort. **D** 1-year mortality of the matched cohort. *CI* confidence interval, *HR* hazard ratio, *NSTE-ACS* non-ST segment elevation acute coronary syndrome, *UFH* unfractionated heparin

In this study, the follow-up was fully completed. In the unmatched study cohort, 275 (34.6%) patients died during a median follow-up of 5.2 years. Over a follow-up period of 30 days, patients with suspected NSTE-ACS and pre-hospital UFH administration exhibited a 30-day mortality rate of 0.4%, compared to 1.4% in those without UFH administration, resulting in an adjusted hazard ratio of 0.28 (95% CI 0.05–1.00, *p* = 0.049, Fig. 2a). No significant difference was observed in 1-year mortality (heparin 5.6% vs. no-heparin 7.7%, adjusted hazard ratio 0.66, 95% CI 0.42–1.05, *p* = 0.078, Fig. 2c).

In the propensity matched cohort, 208 (34.3%) patients died during a median follow-up of 5.2 years. After matching, there was no significant difference in 30-day mortality (heparin 0.4% vs. no-heparin 1.3%, Fig. 2b) and 1-year mortality (heparin 5.5% vs. no-heparin 7.2%, adjusted hazard ratio 0.98, CI 0.95–1.01, *p* = 0.22, Fig. 2d).

Patients with intrahospital confirmed NSTEMI diagnosis and pre-hospital UFH administration did not demonstrate a significant 1-year mortality benefit compared to those without UFH in both unmatched (adjusted hazard ratio 0.88, 95% CI 0.40–1.96, *p* = 0.75) and propensity-matched cohorts (adjusted hazard ratio 0.99, 95% CI 0.90–1.08, *p* = 0.76, Supplementary Fig. 1). Subgroup analysis of UFH recipients, stratified by confirmed versus unconfirmed NSTEMI diagnosis, revealed a non-significant trend toward higher mortality among confirmed NSTEMI patients (unmatched cohort, hazard ratio 2.03, 95% CI 0.95–4.23, *p* = 0.07; propensity matched, hazard ratio 1.04, 95% CI 0.98–1.11, *p* = 0.16, Supplementary Fig. 2).

Bleeding events during the hospital course occurred at a low frequency (< 2%) and were not different between groups (Supplementary Table 1).

## Discussion

In this propensity score matched study of patients with suspected NSTE-ACS, pre-hospital heparin administration was not associated with improved 30-day or 1-year mortality, as compared to those not pre-treated with heparin. Relevant bleeding events were not evident in either of the two groups. These findings challenge the frequent use of pre-hospital parenteral anticoagulation therapy in this patient population, suggesting a potential necessity for a more restrained approach.

### Pre-hospital heparin administration in patients with suspected NSTE-ACS

In the management of ACS, parenteral anticoagulation plays a pivotal role in the initial treatment and is advocated for all patients with ACS upon diagnosis [1, 6, 14]. Despite the absence of guideline recommendations supporting pre-hospital anticoagulation for patients with suspected NSTE-ACS before diagnostic confirmation, these patients are often pretreated with UFH in many countries [1, 5, 6, 9]. In fact, there is practically no evidence regarding pre-hospital heparin or aspirin administration in NSTE-ACS. UFH has historically been the preferred anticoagulant [15]. We observed prehospital UFH administration in 40.8% of patients presenting with suspected NSTE-ACS via emergency service in the emergency department. In these patients, cardiovascular risk factors, such as hypertension, diabetes, hyperlipidemia, or known history of CAD, CKD and stroke, did not appear to be directly linked to the decision for pre-hospital heparin UFH administration. Conversely, patients with prior oral anticoagulation in their premedication less likely received UFH pre-treatment.

The diagnosis of NSTE-ACS in the pre-hospital setting poses a significant challenge, especially in the absence of biomarkers and imaging modalities [1, 9]. Over 30% of ACS patients may have a normal ECG, on the other hand, the presence of ischemic ECG abnormalities is frequent, contributing to an increased likelihood of diagnosing ACS [1, 16–18]. According to the current guidelines, the initial ECG-guided risk stratification should trigger treatment decisions in the pre-hospital setting, influencing factors such as the selection of the target hospital [1, 5, 9]. Especially in cases of suspected NSTE-ACS with high-risk factors, such as recurrent or ongoing chest pain refractory to medical treatment or recurrent dynamic ECG changes suggestive of ischemia, an immediate invasive strategy with emergency angiography is currently recommended [1]. However, even in individuals with elevated risk profiles, the optimal timing for initiating heparin treatment, along with the efficacy and safety in the preclinical setting, remains uncertain [9, 15].

Our observations suggest that approximately 19% of patients with suspected NSTE-ACS had a confirmed NSTEMI diagnosis during their intra-hospital assessment. Potential overtreatment in suspected NSTE-ACS poses several risks. First, the prehospital working diagnosis of NSTE-ACS encounters some differential diagnoses, such as aortic dissection and pericarditis, with potential contraindications for heparin pretreatment. Furthermore, the potential risk of heparin-induced thrombocytopenia is present and is known to correlate with adverse outcomes in ACS patients [19]. Moreover, decisions regarding the initiation of anticoagulation must carefully consider the benefits of antithrombotic therapy weighed against the potential risks of bleeding [1, 20, 21]. Bleeding complications in ACS have been linked to increased mortality in prior studies [22–24]. Notably, our observations following pre-hospital UFH administration showed no significant increase in bleeding events.

In this context, not only the application of risk scores but also the use of point-of-care troponin tests offers potential assistance in prehospital risk assessment and may potentially impact targeted pharmacological therapy in prehospital settings [25–29].

### Association between pre-hospital heparin administration and mortality in NSTE-ACS

Current guidelines recommend the administration of parenteral anticoagulation at the time of confirmed diagnosis for patients with STEMI and NSTE-ACS [1, 7, 8, 30]. For NSTE-ACS patients anticipated to undergo immediate or early invasive angiography and PCI, UFH is the recommended anticoagulant [1]. Studies have also revealed benefits of newer parenteral anticoagulants, such as low molecular weight heparin, over UFH [14, 31, 32]. Specifically, for NSTE-ACS patients not undergoing early invasive angiography within 24 h of diagnosis, fondaparinux is favored over enoxaparin (plus UFH bolus during PCI), as supported by the OASIS-5 trial findings [1, 33].

To the best of our knowledge, no data currently exist demonstrating the advantage of pre-hospital over in-hospital administration of anticoagulants in suspected NSTE-ACS [1, 9]. In this study, pre-hospital UFH administration in suspected NSTE-ACS was not associated with short- and long-term mortality, as compared to those not pre-treated with UFH. This is in line with recent observations in patients undergoing PCI in NSTE-ACS, indicating that parenteral anticoagulation may not reduce the risk of all-cause death or MI [34]. Several explanations may account for the absence of mortality reduction following pre-hospital UFH administration. First, previous analyses, which demonstrated the benefits of early heparin application in clinical settings, were predominantly conducted before the widespread adoption of dual antiplatelet therapy (DAPT) as the standard approach in acute MI [7, 8, 35, 36]. DAPT has markedly improved patient outcomes and is recommended in NSTE-ACS [1, 30, 37]. Secondly, over the past two decades, changes in clinical practice have led to the widespread use of PCI, especially in NSTE-ACS high-risk patients [1]. When combined with the routine use of DAPT, this shift in management strategies may contribute to diminishing the potential protective effect of early anticoagulation therapy. Lastly, only a small proportion of patients with suspected NSTE-ACS had a confirmed MI. These results could be considered predictable, given the neutral outcomes observed in trials investigating pre-treatment with P2Y12 inhibitors in NSTEMI (ACCOAST) and even in STEMI patients (ATLANTIC), who are more likely to have their diagnosis confirmed and receive stent implantation [38, 39]. Observations from these studies also revealed a minimal time difference between pre-treated patients, ranging from approximately 30 min in the ATLANTIC to a few hours in ACCOAST trial [38, 39].

Consequently, these findings challenge pre-hospital parenteral anticoagulation therapy in patients with suspected NSTE-ACS, which is frequently practiced in many countries. Emergency medical services typically spend 15–20 min on scene with this suspected diagnosis, with transport times of 5–20 min in urban areas; thus, pre-hospital heparin administration could save approximately 20–40 min. Particularly in urban settings, where rapid assessment of biomarkers and angiography are readily available for precise risk assessment and revascularization, early heparin administration may offer limited benefit to these patients. Hypothesis-generating, it might potentially warrant a more restrained approach, pending a thorough in-hospital risk assessment. Further research efforts are needed to address the ongoing uncertainty regarding the efficacy and safety of pre-clinical parenteral anticoagulation in patients with suspected NSTE-ACS. As a consequence, our results also call for a formal assessment of the efficacy of administering acetylsalicylic acid to all suspected NSTE-ACS.

### Limitations

The primary limitation of this study lies in its non-randomized design as a single-center cohort study, preventing causal conclusions between treatment and outcomes. To address this limitation, propensity matching was employed, resulting in a high degree of balance among the matched patients. However, the presence of unmeasured variables not included in the propensity matching may introduce bias in treatment allocation and may result in residual confounding.

The data presented here pertain to a cohort with suspected NSTE-ACS presenting via emergency service to the emergency department. Nevertheless, the final diagnosis of NSTEMI was confirmed in only 19% of the cases, limiting the generalizability of our findings to this patient population. In addition, the lack of invasive angiography data constrains the assessment of prehospital heparin's impact on acute coronary status. Furthermore, bleeding events were retrospectively identified exclusively through an ICD-10 code query, potentially leading to an underestimation of bleeding events due to potential coding omissions.

Given these limitations, our findings should be considered hypothesis-generating and first validated in a multicenter registry with higher case numbers. Given that no data currently exist demonstrating the advantage of pre-hospital over in-hospital administration of anticoagulants in suspected NSTE-ACS, and despite its frequent use depending on regional practices, this underscores the imperative for a randomized controlled trial to evaluate prehospital heparin administration in patients with suspected NSTE-ACS.

## Conclusion

In this propensity score matched study, pre-hospital heparin administration in patients presenting with suspected NSTE-ACS to the emergency department was not associated with improved clinical outcome. These findings question the commonly practiced pre-hospital parenteral anticoagulation therapy in this ACS population and might potentially warrant a more restricted utilization pending in-hospital risk assessment.

## Supplementary Information

Below is the link to the electronic supplementary material.

Supplementary file1 (DOCX 2159 KB)

## Abbreviations

ACS: Acute coronary syndrome
CAD: Coronary artery disease
CKD: Chronic kidney disease
ECG: Electrocardiogram
hs-cTnT: High-sensitivity cardiac troponin T
MI: Acute myocardial infarction
NSTE: Non-ST segment elevation
PAD: Peripheral artery disease
PCI: Percutaneous coronary intervention
STEMI: ST-elevation myocardial infarction
UFH: Unfractionated heparin
