# Supplementary material for: Pre-hospital admission of heparin in patients with suspected non-ST segment elevation acute coronary syndrome
Source: Clin Res Cardiol. 2024 Aug 5;114(6):738–48. doi: 10.1007/s00392-024-02507-1 (PMC12089214; doi:10.1007/s00392-024-02507-1)
Supplement: Supplementary file 1 — Supplementary file1 (DOCX 2159 KB) [file 392_2024_2507_MOESM1_ESM.docx]

***SUPPLEMENTARY APPENDIX***

***Supplementary Table 1***. Bleeding events stratified by prehospital UFH administration vs. no prehospital UFH administration.

|  | **No UFH**  **(N=731)** | **UFH**  **(N=503)** |
| --- | --- | --- |
| Acute blood loss anemia No. | 3 | 2 |
| Diverticulitis of the colon with perforation and abscess, without indication of bleeding No. | 0 | 1 |
| Bleeding and hematoma as a complication of a procedure, not classified elsewhere No. | 2 | 1 |
| Diverticulosis of the colon without perforation, abscess, or indication of bleeding No. | 1 | 1 |
| Peptic ulcer, chronic or unspecified, with bleeding No. | 1 | 0 |
| Diverticulitis of the colon without perforation, abscess, or indication of bleeding No. | 1 | 1 |
| Gastrointestinal bleeding, not specified No. | 1 | 2 |
| Bleeding, not classified elsewhere No. | 1 | 0 |

UFH: unfractionated heparin.

***Supplementary figure 1****:* Kaplan–Meier curves of patients with confirmed NSTEMI diagnosis and pre-hospital UFH administration vs. without pre-hospital UFH administration.


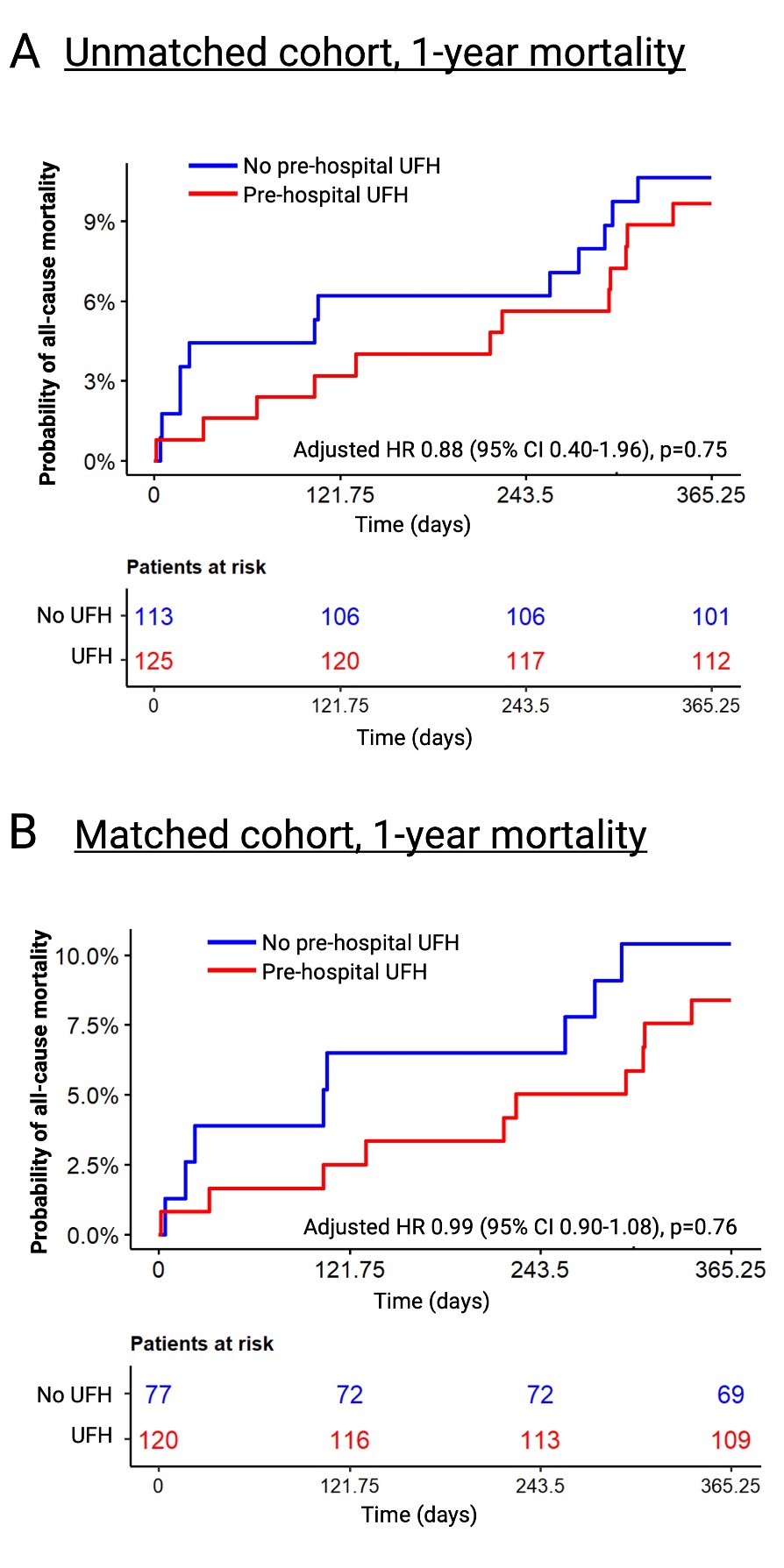


Kaplan–Meier curves of the unmatched and matched study cohort comparing patients with confirmed NSTEMI diagnosis with vs. without pre-hospital UFH. **A**: 1-year mortality of the unmatched cohort. **B**: 1-year mortality of the matched cohort. CI: confidence interval; HR: hazard ratio; NSTEMI: non-ST-elevation myocardial infarction; UFH: unfractionated heparin.

***Supplementary figure 2****:* Kaplan–Meier curves of UFH recipients, stratified by confirmed versus unconfirmed NSTEMI diagnosis.


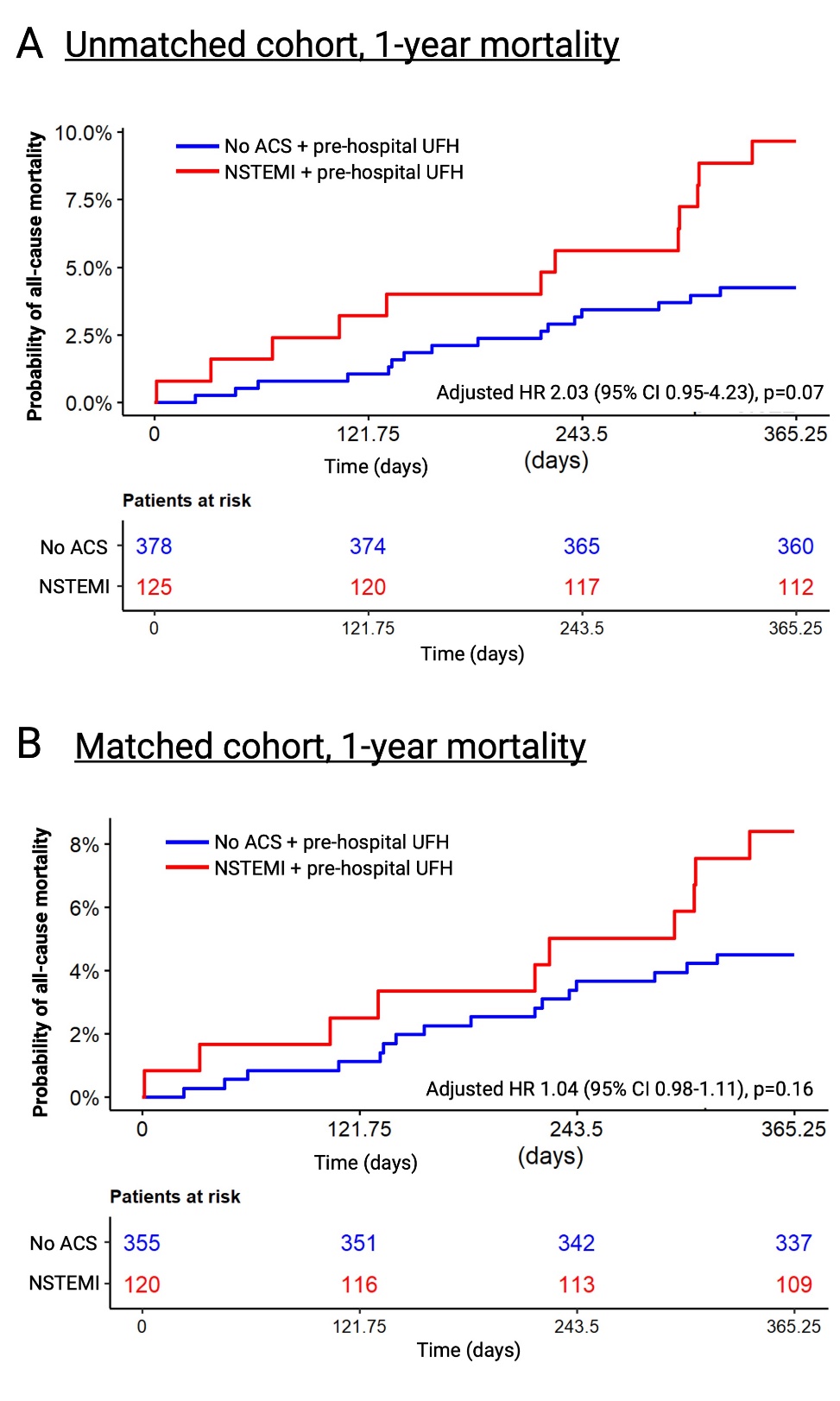


Kaplan–Meier curves of the unmatched and matched study cohort comparing UFH recipients, stratified by confirmed versus unconfirmed NSTEMI diagnosis. **A**: 1-year mortality of the unmatched cohort. **B**: 1-year mortality of the matched cohort. ACS: acute coronary syndrome; CI: confidence interval; HR: hazard ratio; NSTEMI: non-ST-elevation myocardial infarction; UFH: unfractionated heparin.
